# Assessing the patient experience of respiratory syncytial virus infection: development of a patient-reported outcome measure

**DOI:** 10.1186/s12955-022-02066-x

**Published:** 2023-02-28

**Authors:** Carla (DeMuro) Romano, Lyn Finelli, Sandy Lewis, Valerie Williams, Emily Martin, Matthew Phillips, Todd L. Saretsky, Josephine Norquist

**Affiliations:** 1 RTI Health Solutions, 3040 East Cornwallis Road, Post Office Box 12194 Research Triangle Park, Durham, NC 27709-2194 USA; 2 Center for Observational and Real-World Evidence, Merck and Co., Inc, Rahway, NJ USA; 3 School of Public Health, University of Michigan, Ann Arbor, MI USA; 4 Biostatistics and Research Data Sciences, Patient-Centered Endpoints and Strategy Group, Merck and Co., Inc, Rahway, NJ USA

**Keywords:** Respiratory syncytial virus (RSV), RSV Infection, Intensity and Impact Questionnaire (RSV-iiiQ), Symptoms, Impact, Disease burden

## Abstract

**Background:**

Respiratory syncytial virus (RSV) causes significant morbidity and mortality in older adults. Despite a number of RSV vaccine candidates in clinical trials, there are no existing disease-specific, self-reported measures that assess the symptoms and severity of RSV infection from the perspective of adult patients with acute RSV. The objective of this study was to describe the initial conceptualization and development of the RSV Infection, Intensity and Impact Questionnaire (RSV-iiiQ), a new patient-reported outcome measure.

**Methods:**

A targeted review of the literature identified relevant existing measures, symptoms, and impacts of RSV. A draft version of the RSV-iiiQ was developed based on the Influenza Intensity and Impact Questionnaire (Flu-iiQ) with expert input. Qualitative interviews (*N* = 20) were conducted with participants to optimize the RSV-iiiQ conceptual model and confirm the content validity of the RSV-iiiQ. Interviews included concept elicitation and a cognitive debriefing assessment. A draft conceptual framework was developed, and the electronic clinical outcome assessment was piloted. All steps of instrument development followed Food and Drug Administration guidance for patient-reported outcomes.

**Results:**

In-depth concept elicitation interviews followed by cognitive debriefings demonstrated that the content of the items was comprehensive, covered the breadth of RSV symptoms and impacts, and was relevant to the experiences of individuals with RSV. Both the paper and electronic versions of the RSV-iiiQ were easily completed. Minor refinements were made to some items based on participant feedback, and the draft conceptual framework was refined.

**Conclusions:**

The RSV-iiiQ was developed for use in clinical trials to measure the symptom intensity and impact of acute RSV infection from the perspective of adult patients. The tool was developed in accordance with current regulatory guidance and is useful to support patient-focused drug development.

**Supplementary Information:**

The online version contains supplementary material available at 10.1186/s12955-022-02066-x.

## Background

Respiratory syncytial virus (RSV) has been recognized as a cause of significant morbidity and mortality in adults. Approximately 3% to 7% of healthy older adults and 4% to 10% of adults with underlying medical conditions experience RSV infection annually in North America [1–3]. In addition to causing primary upper and lower respiratory tract infection, RSV infection can exacerbate chronic underlying cardiac and pulmonary conditions, and antecedent RSV infection may trigger hospitalization for these conditions. In a typical RSV season, among adults ≥ 65 years of age or older or adults with underlying cardiopulmonary diseases hospitalized with laboratory-confirmed RSV infection, RSV infection accounts for an estimated 11% of hospitalizations for pneumonia, 11% of hospitalizations for chronic obstructive pulmonary disease, 5% of hospitalizations for congestive heart failure, and 7% of hospitalizations for asthma [2, 4]. The economic effects of RSV infection are substantial, with hospitalization costs estimated to be between $1 billion and $5 billion annually in the United States [2, 5]. Absence from work, outpatient or emergency department visits, declines in functional status, and inability to perform daily activities are additional underrecognized costs associated with adult RSV infection [6, 7].

There are a number of RSV vaccine and therapeutic candidates being investigated in clinical trials, but there are no existing clinical outcome assessment measures for RSV symptom severity for use in clinical trials that meet the standards described in the patient-reported outcome (PRO) guidance for medical product labeling from the Food and Drug Administration (FDA) [8]. Given the highly symptomatic nature of RSV infection, patient report is useful to inform treatment benefit of novel vaccine or drug candidates. The FDA 2009 guidance stipulates that any PRO measure referenced in product labeling must be developed with extensive input from patients and be thoroughly tested in the populations involved in the clinical trials. The 2018 FDA draft discussion documentation suggests novel instrument development should be undertaken if an existing measure cannot be used or appropriately modified [9].

The Influenza Intensity and Impact Questionnaire (Flu-iiQ) is a well-developed and carefully evaluated PRO measure used to assess influenza and influenza-like illness symptom severity and other impacts [10], as identified in a literature review. The Flu-iiQ contains items describing symptoms that commonly overlap with RSV infection. Given the similar pathophysiology of influenza and RSV, their common symptoms, and the solid psychometric development of the Flu-iiQ, it was selected as the foundation for a new measure of RSV, the RSV Infection, Intensity and Impact Questionnaire (RSV-iiiQ). The RSV-iiiQ is a PRO measure designed in full accordance with current regulatory standards and industry-accepted best practices to assess the severity of symptoms and impacts of RSV infection from the perspective of adult patients with acute RSV. The RSV-iiiQ incorporates the majority of domains and items (e.g., influenza symptoms, impact on daily activities, impact on emotions) from the Flu-iiQ. Symptoms considered clinically important to describe RSV infection but not included in the Flu-iiQ PRO have been added to the RSV-iiiQ as candidate items; items deemed specific to influenza were removed.

The objective of this paper is to describe the development of the RSV-iiiQ, including background literature and qualitative interviews to inform its content. The intended purpose of the RSV-iiiQ is for administration in clinical investigations of RSV, including outcomes evaluation of clinical trials of vaccines and other pharmaceuticals.

## Methods

Development of the RSV-iiiQ was in accordance with current regulatory guidance and best practices and included a review of the literature and input from experts and patients. A literature review was conducted to determine relevant symptoms and impacts of RSV infection and to identify any existing measures designed to assess the RSV patient experience (see Supplemental Table S1 for the search strategy for the literature review). The first stage included a PubMed database search conducted in December 2017 and restricted to English-language articles that used human participants and were not comments, letters, or editorials and that were published in a 10-year window. Next, desktop research was conducted to identify relevant grey literature, such as conference presentations or government reports focusing on the impact of RSV, including the patient’s ability to perform activities of daily living (ADLs) and psychosocial well-being.

### Expert input

Clinical experts in the field of RSV research participated in several phases on the study. They were United States (US) physicians with experience treating patients with RSV; provided input on the design of the literature review; reviewed and provided recommendations on the draft items and content, the conceptual framework, and patient pilot testing; and served as consultants throughout the study.

### Patient interviews

Two iterative rounds of in-depth interviews were conducted during two RSV seasons (2017–2018 and 2018–2019) to elicit comprehensive descriptions of symptoms and impacts of RSV infection (concept elicitation) as well as to refine the draft RSV-iiiQ item set (cognitive debriefing). Interviews lasted no more than 60 min. Approximately 20 min were allotted for concept elicitation, and 40 min were allotted for cognitive debriefing. The study protocol was reviewed and approved by the University of Michigan Institutional Review Board (Federalwide Assurance No. 4969) and RTI International Institutional Review Board (Federalwide Assurance No. 3331).

Participants were recruited through the University of Michigan (in Ann Arbor, Michigan) and the Global Market Research Group (headquartered in Carlsbad, California). Prospectively identified participants were invited at the time of the study office visit. Clinical sites also reviewed medical records and, within 45 days of the date of diagnosis, invited patients with a positive RSV polymerase chain reaction (PCR) test. Retrospectively identified participants were invited to participate via telephone call or email. Study sites attempted to recruit participants as soon as possible after confirmation of an RSV diagnosis by PCR and when the patient self-reported feeling well enough to participate in the interview. Individuals were eligible to participate if they had acute RSV infection confirmed by a PCR assay within 45 days of being contacted to participate in the qualitative interview. Individuals were excluded if they presented with a comorbid respiratory condition, received supplemental oxygen therapy for a condition other than RSV, had chemotherapy administered in the previous 12 months, or received an investigational medicinal product in the previous 30 days. Informed consent was obtained from each participant prior to the interview.

Interviews were conducted using standardized qualitative research methods [11, 12] and began with open-ended questions to ascertain participants’ experiences with RSV and its impact on their lives. These general questions were followed by targeted probes intended to evaluate the course of RSV symptoms and what symptoms were considered the most bothersome or concerning. Following this concept elicitation component, the draft RSV-iiiQ items were reviewed with participants for their input and endorsement of symptom and impact concepts included in the instrument. During this cognitive debriefing portion of the interview, participants were asked to “think aloud” by describing their thought processes as they reviewed the draft RSV-iiiQ items. Participants were also asked to provide feedback to confirm the relevance of each item and identify any problems with item wording or response options. Follow-up questions were also asked to help better understand how participants interpreted and responded to each of the draft RSV-iiiQ items.

All interviews were audio recorded, transcribed, and verified for accuracy. Analysis of the qualitative data was conducted using the typed transcripts, and detailed field notes were collected during the interviews. Thematic analysis was conducted through the identification of dominant concepts in each interview, which were compared across other interviews to generate themes or patterns in the ways participants described their experiences [12]. Participants also provided feedback on draft items. Results incorporated representative participant quotes, selected to illustrate participants’ perspectives using their own words. Concept saturation (i.e., the point at which no new information is captured [13]) was documented using a saturation grid.

## Results

### Literature review and identification of an existing measure

A total of 78 abstracts were identified via a PubMed search and reviewed; 16 full-text articles were selected for further review if the identified abstract noted symptoms or patient impacts of RSV. Six articles provided detailed information on specific symptoms, impacts, and experiences associated with RSV in adult patients [3, 14–18]. Desktop research identified nine additional articles or websites [2, 5, 6, 19–24]. Table 1 details all signs, symptoms, and impacts of RSV infection identified in the literature, including impact on daily activities and emotions contained in the Flu-iiQ.

**Table 1:** Signs, symptoms, and impact of RSV identified in the literature

**RSV signs and symptoms and patient experiences**	**Literature source**																**RSV-iiiQ**
	**1**	**2**	**3**	**4**	**5**	**6**	**7**	**8**	**9**	**10**	**11**	**12**	**13**	**14**	**15**	**Total**^** a**^	
**Respiratory symptoms**																	
Coryza (catarrhal inflammation of the mucous membrane in the nose)										x						1	
Cough	x	x		x	x	x	x	x	x	x	x	x	x	x	x	14	x (and cough with phlegm)
Crackles		x														1	
Croup-like cough														x		1	
Dyspnea (difficulty breathing, shortness of breath)	x	x	x	x	x		x		x	x	x	x	x	x	x	13	x
Nasal congestion	x		x	x	x	x		x	x	x				x	x	10	x
Orthopnea												x				1	
Rales	x		x		x											3	
Rhinitis												x				1	
Rhinorrhea	x	x			x		x				x					5	
Rhonchi		x	x													2	
Sputum production			x		x				x			x			x	5	
Wheezing	x	x	x	x	x	x	x	x	x		x	x		x		12	x
**Infections**																	
Bacteremia			x													1	
Bronchiolitis														x		1	
**Pain**																	
Chest pain	x													x		2	
Chills										x						1	
Ear pain		x				x		x								3	
Headache					x	x		x			x			x		5	x
Muscle aches								x								1	x (body aches and pains)
Myalgias							x			x		x		x		4	x (body aches and pains)
Sinus pain		x				x										2	
Nausea/abdominal pain					x											1	
Sore throat/pharyngitis	x	x	x	x	x	x	x	x		x	x	x		x	x	13	x
**Systemic symptoms**																	
General systemic symptoms not described (group of symptoms that can affect many different systems of the body)	x				x											2	
Fatigue								x								1	x
Lethargy/malaise									x	x						2	
Weakness									x							1	
**Fever-related symptoms**																	
Febrile seizures														x		1	
Fever or feverishness	x	x	x		x	x	x	x	x	x	x	x	x	x	x	14	x
Rigors (a sudden feeling of cold with shivering accompanied by a rise in temperature, often with copious sweating, especially at onset or height of a fever)												x				1	
Sweating										x						1	
**Other, including symptoms and impacts**																	
Hoarseness	x				x					x					x	4	
Vomiting														x		1	
Work/training absence				x		x										2	
Diarrhea														x		1	
Interrupted sleep ^b^																0	x
Loss of appetite ^b^																0	x
Hospitalization due to RSV	x^c^	x	x^c^					x	x			x^c^	x			7	
ICU									x				x			2	
Ward confinement				x												1	
Inability to complete ADLs															x	1	x
Inability to get out of bed															x	1	x
Prepare meals/Get your own food ^b^																0	x
Perform your usual activities ^b^																0	x
Leave the home ^b^																0	x
Concentrate on tasks ^b^																0	x
Take care of yourself ^b^																0	x
Walk up a flight of stairs ^b^																0	x
Dress yourself ^b^																0	x
Irritable ^b^																0	x
Helpless ^b^																0	x
Frustrated ^b^																0	x
Worried ^b^																0	x

*ADL* Activity of daily living, *ICU* Intensive care unit, *RSV* Respiratory syncytial virus, *RSV-iiiQ* RSV Infection, Intensity and Impact Questionnaire

^a^Total number of identified resources mentioning the specific sign or symptom

^b^Additional ADLs and emotional impacts identified in the influenza literature[10, 25, 26]

^c^Study sample was defined as individuals who were hospitalized

Sources: 1, Walsh et al. [27]; 2, Dowell et al. [28]; 3, Falsey et al. [2]; 4, O'Shea et al. [29]; 5, Falsey and Walsh [4]; 6, Hall et al. [7]; 7, Branche and Falsey [1]; 8, Sundaram et al. [17]; 9, Volling et al. [3]; 10, Wald et al. [30]; 11, Li et al. [14]; 12, Uckay et al. [18]; 13, Loubet et al. [15]; 14, Saxena et al. [16]; 15, Falsey et al. [2]

Early symptoms of RSV are nasal congestion and rhinorrhea, cough, shortness of breath, and wheezing. Healthy adults with RSV reported less fever and dyspnea than healthy adults with influenza, although hospitalized adults with RSV reported more wheezing than patients with influenza [22]. As RSV progresses or becomes more severe, patients may experience severe cough, wheezing, or rapid breathing and progressive outcomes of pneumonia or bronchiolitis [31]. RSV symptoms are similar to those of influenza [27], making the two diseases sometimes difficult to differentiate. RSV diagnostic testing is not widely performed in adults because there is a lack of effective therapies [17, 27]. However, influenza often shows annual variability, whereas RSV tends to be more consistent in terms of attack rates and severity [2]. Falsey et al. [2] identified, over the course of four consecutive seasons, RSV infections in cohorts of community-dwelling elderly, high-risk adults, and persons hospitalized with acute cardiopulmonary conditions in Rochester, New York. High-risk patients reported greater functional impairment than healthy elderly patients, with 45% of high-risk individuals reporting an inability to complete ADLs, compared with 39% of healthy elderly patients. For example, inability to get out of bed was reported by 7% of healthy elderly patients and by 25% of those considered to be at high risk.

No RSV-specific measures for use in adults were found in the literature, although Falsey et al. [2] used a set of ADL scales to evaluate functional status in adults with RSV. The Flu-iiQ, a previously developed and psychometrically tested PRO measure for the assessment of symptom severity in influenza and influenza-like illness, was identified as a measure used in clinical trials for influenza vaccine [10, 32]; it was developed using concept mapping with patients and a validity-driven approach with a strong measurement model. The Flu-iiQ covers concepts related to anxiety and depression, such as irritableness, helplessness, frustration, and worry. A number of ADLs are included in the Flu-iiQ, such as getting out of bed, preparing meals, performing usual activities, leaving home, concentrating on tasks, and taking care of oneself. Content analysis indicated that it contains symptoms, ADLs, and psychosocial consequences that are relevant to RSV infection. Given that the pathophysiology of influenza and RSV are similar, we used the Flu-iiQ [10] as a framework for further development of the RSV-iiiQ. The Flu-iiQ was used with permission of Richard Osbourne and Measured Solutions for Health P/L.

### Development of the RSV-iiiQ

RSV symptoms and impacts were mapped on to the content of the Flu-iiQ [9]. Subsequently, clinical experts utilized the results of the literature review and symptoms considered clinically important to describe RSV infection but not included in the Flu-iiQ to construct candidate RSV-iiiQ items. These items were created based on appropriate item-generation principles requiring single-barreled questions, patient-centric language, and ability to change over the course of a clinical study. Specifically, items were added to assess runny nose, wheezing, cough with phlegm, and shortness of breath, as well as two supplementary ADLs: walking up a flight of stairs and dressing oneself. Conversely, the Flu-iiQ items addressing neck pain and impact on others were not retained for the proposed RSV-iiiQ; they were deemed irrelevant to the evaluation of RSV infection based on clinical input and published literature. The initial draft version of the RSV-iiiQ was shared with five RSV clinical experts for their review of the consistency of item content with the clinical syndrome and for preparation and formatting of the tool as a web-based electronic clinical outcome assessment (eCOA).

The initial draft version of the RSV-iiiQ consisted of 29 items evaluating the symptoms, ADLs, and psychosocial impact of RSV infection. For questions related to RSV symptoms (*n* = 17) and psychosocial consequences (*n* = 4), participants were asked to recall the past 24 h and select from a 4-point verbal response scale ranging from “none” to “severe”; for questions about ADLs (*n* = 8), participants were asked to recall the past 24 h and select from a 4-point verbal response scale that ranged from “no difficulty” to “severe difficulty.”

### Participant characteristics

A total of 20 adults participated in semistructured interviews, which utilized a split interview technique that involved concept elicitation followed by a thorough cognitive debriefing of the draft RSV-iiiQ. Participants ranged in age from 26 to 78 years (average age, 52 years), and the majority of the study sample was White (*n* = 15; 75%) and female (*n* = 14; 70%) (Table 2). More than half of participants (65%) had a college degree; 20% reported a high school education only. The majority of respondents (60%) were employed at least part-time.

**Table 2:** Characteristics of interview participants

Characteristic	*N* = 20
Median age (range), years	52 (26–78)
Gender, n (%)	
Male	6 (30)
Female	14 (70)
Race/ethnicity, n (%)	
Asian	1 (5)
Black	4 (20)
Hispanic	0 (0)
White	15 (75)
Education, n (%) ^a^	
Less than high school	0 (0)
High school or equivalent (e.g., GED)	2 (10)
Some college	4 (20)
College degree	8 (40)
Professional or advanced degree	5 (25)
Employment, n (%) ^b^	
Employed full-time	10 (50)
Employed part-time ^c^	2 (10)
Retired	5 (25)
Volunteer	0 (0)
Unemployed	1 (5)
Self-reported RSV symptom severity, n (%)	
Mild	0 (0)
Moderate	14 (70)
Severe	6 (30)

*GED* General equivalency diploma, *RSV* Respiratory syncytial virus

^a^Information on education level was missing for one participant

^b^Information on employment status was missing for two participants

^c^One participant reported working part-time for 20 h per week. The number of hours per week for the other participant is missing

### Concept elicitation

The most common symptoms reported were cough (100%) and fatigue (85%). Shortness of breath, stuffy nose, runny nose, body aches and pains, interrupted sleep, sore throat, wheezing, fever, mucous production, and/or headache were all reported by at least 50% of respondents (Table 3). Time from symptom development to seeking care ranged from 2 to 3 days for those experiencing more severe symptoms and from 1 to 2 weeks for those whose symptoms were less severe. Participants with greater symptom severity expressed more concern about their RSV diagnosis than those with less symptom severity. Participants reported that symptoms subsided over time, noting that cough, breathing difficulties, and runny and/or stuffy nose were the last symptoms to resolve. All participants noted that activity exacerbated their symptoms. The most bothersome symptoms reported by participants included cough, fatigue, and shortness of breath. Symptoms that were most concerning to participants included bronchospasm, difficulty breathing, progression to pneumonia, and a reduced activity level that is “difficult to recover from.”

**Table 3:** Representative participant descriptions of RSV symptoms

**Cough (includes descriptions of mucous production)**
*I had a lot of coughing, especially when I’d lay on my back. And I was coughing up quite a bit of phlegm*
*It started with chest congestion, coughing, lots of movement within the lungs, rattling in the lungs*
**Fatigue/tiredness/exhaustion**
*Yes, I was exhausted. Very exhausted, very tired, and very…yeah, very sleepy, very tired, have to sleep all the time. I couldn’t watch a program without dozing off at that period of time*
*I felt quite tired and sleepy and weak*
**Shortness of breath/difficulty breathing/shallow breathing**
*Like the shortness of breath coming with it, like…I’ll just go out, you know, just doing basic stuff that I could usually do; I just get really short of breath. And it feels like I’ve run a mile, you know, just taking the trash out*
*The shortness of breath came when I would do the stairs or something like that. Or if I would bend over, you know. That never happened. It would get short, something like that*
**Stuffy nose/nasal congestion**
*I still get the stuffed nose*
*It was [nasal] congestion as well*
**Runny nose**
*I was constantly blowing my nose. I would blow, and then the next 5 min I have to do it again. It was really coming*
*Then I started having sinus problems and sniffles…The whole time in the hospital and for days when I came home, for 3 or 4* days* [I had a runny nose]*
**Body aches and pains**
*A lot of body aches all over, but more so in my legs than anywhere else*
*Body aches and pains were definitely present*
**Interrupted sleep**
*Well when I first got it, I was not sleeping very good because of all the coughing, and so I would sleep maybe half an hour or so and then I’d start coughing again and be awake for another half an hour*
*I wake up in the middle of the night. That was one of the things, too. I haven’t been able to go to sleep too well. I just wake up and start coughing*
**Sore throat**
*A really raw, sore throat. It wasn’t like a typical sore throat. It was just the entire throat area was just very irritated*
*And so I started off with head congestion and a super, super sore throat. So I thought that I might have strep*
**Wheezing**
*And not only was I wheezing, but you could hear squeaking, a squeaking noise. I really was having a lot of trouble just breathing*
*Yes. Oh, the wheezing, yes, it’s awful. I feel like I’m, you know, I’m 85*
**Fever/feeling feverish**
*One night I woke up about 3 am and had an almost 102 [degree] fever*
*About 3:00 in the afternoon, I felt a fever spike. And then I had really bad chills and shakes*
**Headache**
*I woke up with a migraine headache for a couple days…very constant. It went from a 10 in the beginning, out of 10, to about a 2 or 3; but it never clearly went away*
*Mild headache. But I just always thought the headache was due to the cough*
**Other symptoms**
*Then it was just really the congestion and the ears. I just remember the ****ears being so stuffed up****. I was concerned about being on a plane [due to ear pain and congestion]*
*And I just ****felt really weak****…And I felt ****hot**** and I felt dehydrated or something. ****My appetite had gone***

*RSV* Respiratory syncytial virus

Participants also detailed both the functional and the emotional impact of RSV infection (Table 4 and Table 5). The most commonly reported functional impact was an inability to participate in sports, exercise, or do other things for enjoyment (45%). Additionally, 8 of the 12 working participants reported missing work or school due to RSV infection. Other functional and emotional impacts reported by at least 20% of respondents included difficulty with self-care activities (e.g., dressing, bathing, preparing meals), leaving home, concentration, a decrease in productivity, and a negative impact on relationships with others. The predominant emotional impacts reported were worry (50%) and frustration (40%).

**Table 4:** Representative participant descriptions of functional impacts on daily activity from RSV

**Getting out of bed**
*I would have to sit on the side of the bed for a while and then get my feet up and then sit upright in the bed for a while before I…It felt better when I leaned forward. It seems like I could, can breathe better when I’m leaning forward. It feels like my lungs could expand better*
*Yeah, I just forced myself to do it [get out of bed]*
**Self-care, meals, and usual activities**
*I can’t do anything. I can hardly make meals. I can’t get up the stairs. I have to crawl up the stairs now*
*I haven’t taken a shower since Saturday morning. It’s too much effort to get in the tub. I just lay in bed, so I’m not really dirty. But I brush my teeth, and I go to the bathroom. I don’t have any energy to stand up in the shower. I would like a bath; but once I get in there, it’s too hard to get out, so…*
**Leaving home**
*I can’t get to the grocery store. I can’t drive my car*
*I don’t want to leave the home. I really don’t want to go anywhere*
**Ability to concentrate**
*It was very foggy. I couldn’t concentrate on anything*
*It takes a lot to do stand-up [comedy]. And then you have to think, and I was messed up. I couldn’t even think of a good joke, you know*
**Using stairs**
*I can’t get up the stairs. I have to crawl up the stairs now*
*[Limited to] once or twice a day, and that’s it. It takes everything out of me to go up. A little less coming down, but I only go up twice*
**Missing work/school**
Yes, I did [miss work]. I missed a few shows because I wasn’t able to do it. I was in bad shape
*My doctor did write me out for the entire week this week…So I would say 2* weeks
**Other daily activity/functional impacts**
*Yes, because I couldn’t exercise at all*
*I mean I kind of try to keep my engaging with other people to kind of a minimum [to avoid spreading RSV]*

*RSV* Respiratory syncytial virus

**Table 5:** Representative participant descriptions of emotional impacts from RSV

**Irritable**
*Tired and short tempered and very impatient, for real*
*Agitated and don’t really feel like being bothered*
**Helpless**
*Like, how am I going to fix this? I think it was really bad. It set me back a few paces*
**Frustrated**
*And so I think the panic was there. And then just the frustration of not being able to stop anything and the length of the illness was significant*
*Um, mainly frustration. I’m just kind of, you know, not being able to, you know, do my work the same as I usually would; not being able to, you know, go to see my son like I usually would or, you know, go to, you know, my daughter’s concert like I would have*
**Worried**
*[Fear] that I was going to die*
*I had a little breakdown. I cried a little bit because I was starting to really get a little bit, afraid that…What if I don’t come out of this?*
*Yeah, [worried about] financial implications. I haven’t been able to work*

*RSV* Respiratory syncytial virus

**Fig. 1:**
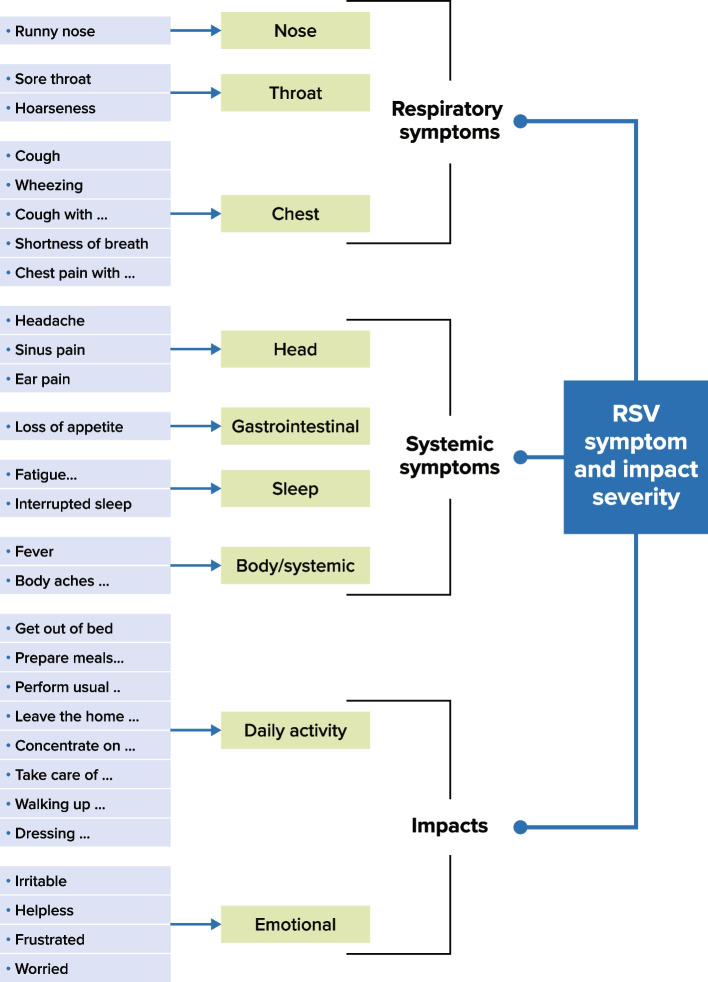
Conceptual framework of RSV-iiiQ. RSV = Respiratory syncytial virus; RSV-iiiQ = RSV Infection, Intensity and Impact Questionnaire

From the combined evidence gathered to support the development of the RSV-iiiQ, a conceptual framework was created (Fig. 1). A conceptual framework describes the expected relationships of items within a domain and the expected relationships among domains within a PRO concept [33]. Our conceptual framework was developed predominantly from patient input but also in part from analysis of dominant themes discovered in the literature and through consultation with clinicians.

### Cognitive debriefing

Participants fully endorsed inclusion of all symptom and impact concepts included in the RSV-iiiQ. Interpretation of the symptom and impact concepts was consistent with those previously described during concept elicitation. Overall, participants found the instructions clear and easy to understand. Participants noted that the 24-h recall period was an appropriate amount of time to consider and report RSV symptoms and impacts, given the fluctuation in RSV symptoms that can occur over a short period of time. Participants found the 4-point categorical response scale ranging from “none” to “severe” to be appropriate for evaluation of RSV symptom severity. Overall endorsement of the item concepts was consistent across all participants. No further changes were made to the instructions, recall period, or response options of items following the conclusion of the round 2 interviews.

The eCOA version of the RSV-iiiQ was a web-based application that could be accessed from computers, tablets, or smartphones. Overall, participants were receptive (and most noted a preference) to using an electronic format to complete the RSV-iiiQ. All participants understood how to access the eCOA’s web-based questionnaire, navigate through the screens, select and change (if needed) their answer, and advance to subsequent questions. Most found the system to be intuitive and required little to no instruction from the interviewer.

### Concept saturation

Concept saturation was assessed throughout the qualitative interview process. All symptoms and consequences contained within the RSV-iiiQ were endorsed by participants during both the initial concept elicitation and again during cognitive debriefing (see Table S2, Supplemental Material). The majority of symptoms were identified following completion of the eighth interview and further endorsed during subsequent interviews. Saturation of emotional consequences occurred following the completion of the tenth interview.

## Discussion

The RSV-iiiQ was designed to capture RSV symptom severity and the emotional and psychosocial impact of RSV infection most commonly experienced by adults. To our knowledge, at the time of this publication, this is the only RSV severity and impact measure developed in accordance with the FDA PRO guidance recommendations. Concepts for inclusion in the RSV-iiiQ were based on the scientific literature, endorsed by clinical experts, and consistent in frequency and terminology with RSV patients’ experience. Results of the participant interviews supported the ability of the items to accurately reflect participants’ perceptions of symptom severity and impact.

This self-completed measure was developed in alignment with the FDA’s 2009 PRO guidance [8] and 2018 draft discussion documentation [9] in support of the modification of an existing measure for an expanded context of use. The overlap in the patient experiences of RSV and influenza facilitated the adaptation of the Flu-iiQ. The supplemental work in the field of RSV adds significantly to the weight of evidence supporting this new tool in this specific context of use. Notably, the use of PRO measures in clinical trials aligns with regulatory goals to promote patient-focused drug development [34], a systematic approach to incorporating patient needs into drug development to support novel treatments with outcomes meaningful to patients. Finally, the older adult participants (those aged 60 or older) were receptive to using an electronic format to complete the RSV-iiiQ and were able to access the measure and read and respond to the items presented either on a laptop, a tablet, or a smartphone. Availability of both paper and electronic versions of the tool will contribute to the accessibility of the RSV-iiiQ across this patient population.

Limitations of this study include the potential differences between the qualitative sample and the clinical trial population regarding the time between infection and diagnosis, the use of convenience sampling, and the development of the instrument in a US population only. Cross-cultural adaptation and translation would be required for use in other languages and/or cultures. Rigorous psychometric evaluations should be conducted to support key measurement properties, including the RSV-iiiQ’s reliability, validity, and ability to detect change and the development of preliminary responder definitions.

## Conclusions

The patient voice is essential to inform clinical trials and is useful in evaluations of RSV vaccine efficacy. To date, there are no clinical outcome assessment measures to assess RSV-associated symptoms or ADLs that include the patient perspective. The RSV-iiiQ was developed for use in clinical trials to measure RSV symptom intensity and impact in adults with acute RSV. A targeted literature review, expert input, and patient interviews confirmed that the RSV-iiiQ considers the symptoms, ADLs, and psychosocial items that are relevant to patients with RSV infection. 

## Supplementary Information

**Additional file 1.** **Table S-1.** PubMed search criteria: database: PubMed (search conducted 11 December 2017). **Table S-2** Concept elicitation (Saturation) grid.

## Data Availability

The datasets generated and/or analyzed during the current study are included in this published article and its supplementary information files.

## Abbreviations

ADL: Activity of daily living
eCOA: Electronic clinical outcome assessment
FDA: Food and Drug Administration
Flu-iiQ: Influenza Intensity and Impact Questionnaire
GED: General equivalency diploma
ICU: Intensive care unit
MeSH: Medical Subject Headings
PRO: Patient-reported outcome
RSV: Respiratory syncytial virus
RSV-iiiQ: RSV Infection, Intensity and Impact Questionnaire
US: United States

## References

[1] Branche AR, Falsey AR (2015). Respiratory syncytial virus infection in older adults: an under-recognized problem. Drugs Aging.

[2] Falsey AR, Hennessey PA, Formica MA, Cox C, Walsh EE (2005). Respiratory syncytial virus infection in elderly and high-risk adults. N Engl J Med.

[3] Volling C, Hassan K, Mazzulli T, Green K, Al-Den A, Hunter P (2014). Respiratory syncytial virus infection-associated hospitalization in adults: a retrospective cohort study. BMC Infect Dis.

[4] Falsey AR, Walsh EE (2000). Respiratory syncytial virus infection in adults. Clin Microbiol Rev.

[5] Pastula ST, Hackett J, Coalson J, Jiang X, Villafana T, Ambrose C (2017). Hospitalizations for respiratory syncytial virus among adults in the United States, 1997–2012. Open Forum Infect Dis..

[6] Widmer K, Griffin MR, Zhu Y, Williams JV, Talbot HK (2014). Respiratory syncytial virus- and human metapneumovirus-associated emergency department and hospital burden in adults. Influenza Other Respir Viruses.

[7] Hall CB, Long CE, Schnabel KC (2001). Respiratory syncytial virus infections in previously healthy working adults. Clin Infect Dis.

[8] Food and Drug Administration (FDA). Guidance for industry patient-reported outcome measures: use in medical product development to support labeling claims. 2009. Available at: https://www.fda.gov/media/77832/download. Accessed 2 Sept 2020. 10.1186/1477-7525-4-79 PMC1629006 17034633

[9] Food and Drug Administration. Patient-focused drug development (PFDD) guidance: methods to identify what is important to patients & select, develop or modify fit-for-purpose clinical outcomes assessments. Attachment to guidance 3 discussion document – appendices. 2018. https://www.fda.gov/media/116281/download. Accessed 2 Sept 2020.

[10] Osborne RH, Norquist JM, Elsworth GR, Busija L, Mehta V, Herring T (2011). Development and validation of the Influenza Intensity and Impact Questionnaire (FluiiQ). Value Health..

[11] Willis G. Cognitive interviewing: a “how to” guide. 1999. Paper read at Annual Meeting of the American Statistical Association, at Research Triangle Park, Research Triangle Institute, NC.

[12] Boeije HA (2002). Purposeful approach to the constant comparative method in the analysis of qualitative interviews. Qual Quant.

[13] Guest G, Bunce A, Johnson L (2006). How many interviews are enough?. Field Methods.

[14] Li L, Avery R, Budev M, Mossad S, Danziger-Isakov L (2012). Oral versus inhaled ribavirin therapy for respiratory syncytial virus infection after lung transplantation. J Heart Lung Transplant.

[15] Loubet P, Lenzi N, Valette M, Foulongne V, Krivine A, Houhou N (2017). Clinical characteristics and outcome of respiratory syncytial virus infection among adults hospitalized with influenza-like illness in France. Clin Microbiol Infect.

[16] Saxena S, Singh D, Zia A, Umrao J, Srivastava N, Pandey A (2017). Clinical characterization of influenza A and human respiratory syncytial virus among patients with influenza like illness. J Med Virol.

[17] Sundaram ME, Meece JK, Sifakis F, Gasser RA, Belongia EA (2014). Medically attended respiratory syncytial virus infections in adults aged >/= 50 years: clinical characteristics and outcomes. Clin Infect Dis.

[18] Uckay I, Gasche-Soccal PM, Kaiser L, Stern R, Mazza-Stalder J, Aubert JD (2010). Low incidence of severe respiratory syncytial virus infections in lung transplant recipients despite the absence of specific therapy. J Heart Lung Transplant.

[19] Bracht M, Basevitz D, Cranis M, Paulley R (2011). Impact of respiratory syncytial virus: the nurse's perspective. Drugs R D.

[20] Diez-Domingo J, Perez-Yarza EG, Melero JA, Sanchez-Luna M, Aguilar MD, Blasco AJ (2014). Social, economic, and health impact of the respiratory syncytial virus: a systematic search. BMC Infect Dis.

[21] Fleming DM, Taylor RJ, Lustig RL, Schuck-Paim C, Haguinet F, Webb DJ (2015). Modelling estimates of the burden of respiratory syncytial virus infection in adults and the elderly in the United Kingdom. BMC Infect Dis.

[22] National Foundation for Infectious Diseases. RSV in older adults: a hidden annual epidemic (September 2016). 2016. Available at: http://www.nfid.org/publications/reports/rsv-report.pdf. Accessed 15 Feb 2019.

[23] Walsh EE, Falsey AR (2012). Respiratory syncytial virus infection in adult populations. Infect Disord Drug Targets.

[24] Karron RA, Black RE (2017). Determining the burden of respiratory syncytial virus disease: the known and the unknown. Lancet.

[25] Yang J, Jit M, Zheng Y, Feng L, Liu X, Wu J (2017). The impact of influenza on the health related quality of life in China: an EQ-5D survey. BMC Infect Dis..

[26] Gozalo PL, Pop-Vicas A, Feng Z, Gravenstein S, Mor V (2012). Effect of influenza on functional decline. J Am Geriatr Soc.

[27] Walsh EE, Peterson DR, Falsey AR (2007). Is clinical recognition of respiratory syncytial virus infection in hospitalized elderly and high-risk adults possible?. J Infect Dis.

[28] Dowell SF, Anderson LJ, Gary HE, Erdman DD, Plouffe JF, File TM (1996). Respiratory syncytial virus is an important cause of community-acquired lower respiratory infection among hospitalized adults. J Infect Dis.

[29] O'Shea MK, Ryan MA, Hawksworth AW, Alsip BJ, Gray GC (2005). Symptomatic respiratory syncytial virus infection in previously healthy young adults living in a crowded military environment. Clin Infect Dis.

[30] Wald TG, Miller BA, Shult P, Drinka P, Langer L, Gravenstein S (1995). Can respiratory syncytial virus and influenza A be distinguished clinically in institutionalized older persons?. J Am Geriatr Soc.

[31] Mayo Clinic. Thrombocytopenia (low platelet count). 2017. Available at: https://www.mayoclinic.org/diseases-conditions/thrombocytopenia/symptoms-causes/syc-20378293. Accessed 29 Mar 2018.

[32] van Essen GA, Beran J, Devaster JM, Durand C, Duval X, Esen M (2014). Influenza symptoms and their impact on elderly adults: randomised trial of AS03-adjuvanted or non-adjuvanted inactivated trivalent seasonal influenza vaccines. Influenza Other Respir Viruses.

[33] Donatti C, Wild D, Hareendran A. The use of conceptual models, conceptual frameworks, and endpoint models to support label claims of treatment benefit using patient reported outcomes. ISPOR Connections. 2008;14:9–12.

[34] Food and Drug Administration. FDA patient-focused drug development guidance series for enhancing the incorporation of the patient’s voice in medical product development and regulatory decision making. 2019. Available at: https://www.fda.gov/drugs/development-approval-process-drugs/fda-patient-focused-drug-development-guidance-series-enhancing-incorporation-patients-voice-medical. Accessed 26 Mar 2020.

