# Supplementary material for: Assessing the patient experience of respiratory syncytial virus infection: development of a patient-reported outcome measure
Source: Health Qual Life Outcomes. 2023 Feb 28;21:20. doi: 10.1186/s12955-022-02066-x (PMC9976435; doi:10.1186/s12955-022-02066-x)
Supplement: Supplementary file 1 — Additional file 1. Table S-1. PubMed search criteria: database: PubMed (search conducted 11 December 2017). Table S-2 Concept elicitation (Saturation) grid. [file 12955_2022_2066_MOESM1_ESM.docx]

# Supplementary Material

## Table S-1. PubMed Search Criteria: Database: PubMed (Search Conducted 11 December 2017)

| Search No. | Search Terms | No. of Articles |
| --- | --- | --- |
| Disease | | |
| #1 | “Respiratory Syncytial Virus Infections”[Majr] OR “respiratory syncytial virus infection”[Title/Abstract] OR “respiratory syncytial virus infections”[Title/Abstract] OR RSV infection*[Title/Abstract] | 3,017 |
| Symptoms | |  |
| #2 | #1 AND (“Signs and Symptoms”[Majr] OR sign[Title] OR signs[Title] OR symptom*[Title/Abstract] OR impact*[Title/Abstract] OR burden*[Title/Abstract] OR clinical*[Title/Abstract]) | 1,271 |
| Qualitative studies | |  |
| #3 | #2 AND (“Qualitative Research”[MeSH] OR “Focus Groups”[MeSH] OR “Interviews as Topic”[MeSH] OR “Patient Reported Outcome Measures”[MeSH] OR “Self Report”[MeSH] OR “Outcome Assessment (Health Care)”[MeSH] OR “Surveys and Questionnaires”[MeSH] OR qualitativ*[Title/Abstract] OR focus group*[Title/Abstract] OR interview*[Title/Abstract] OR oral histor*[Title/Abstract] OR self report*[Title/Abstract] OR patient report*[Title/Abstract] OR proxy report*[Title/Abstract] OR patient perspective*[Title/Abstract] OR patient’s perspective*[Title/Abstract] OR patients’ perspective*[Title/Abstract] OR questionnaire*[Title/Abstract] OR survey*[Title/Abstract] OR diary[Title/Abstract] OR assessment*[Title/Abstract] OR instrument*[Title/Abstract] OR “Interview”[Publication Type]) | 320 |
| Exclusions | |  |
| #4 | “Animals”[MeSH] NOT “Humans”[MeSH] | 1,049,892 |
| #5 | “Comment”[Publication Type] OR “Letter”[Publication Type] OR “Editorial”[Publication Type] | 653,297 |
| #6 | (“Child”[MeSH] OR child*[Title/Abstract] OR pediatric*[Title/Abstract] OR paediatric*[Title/Abstract] OR “Adolescent”[MeSH] OR adolescen*[Title/Abstract] OR teen*[Title/Abstract] OR youth[Title/Abstract] OR “Infant”[MeSH] OR infant*[Title/Abstract] OR newborn*[Title/Abstract]) NOT (“Adult”[MeSH] OR adult*[Title/Abstract] OR elder*[Title/Abstract] OR senior citizen*[Title/Abstract] OR middle age*[Title/Abstract]) | 576,664 |
| Total | |  |
| #7 | (#3 NOT (#4 OR #5 OR #6)) | **78** |

MeSH = Medical Subject Heading; RSV = respiratory syncytial virus.

Limits: December 2007-Present; English; Humans; No Comments, Letters, Editorials

## Table S-2. Concept Elicitation (Saturation) Grid

| Concept | Participants | | | | | | | | | | | | | | | | | | | | Total (N = 20) |
| --- | --- | --- | --- | --- | --- | --- | --- | --- | --- | --- | --- | --- | --- | --- | --- | --- | --- | --- | --- | --- | --- |
|  | 1 | 2 | 3 | 4 | 5 | 6 | 7 | 8 | 9 | 10 | 11 | 12 | 13 | 14 | 15 | 16 | 17 | 18 | 19 | 20 |  |
| Symptoms | | | | | | | | | | | | | | | | | | | | | |
| Cough | ✓ | ✓ | ✓ | ✓ | ✓ | ✓ | ✓ | ✓ | ✓ | ✓ | ✓ | ✓ | ✓ | ✓ | ✓ | ✓ | ✓ | ✓ | ✓ | ✓ | 20 |
| Fatigue/tiredness/exhaustion | ✓ | ✓ | ✓ | ✓ | ✓ | ✓ | ✓ | ✓ |  | ✓ | ✓ | ✓ | ✓ | ✓ |  | ✓ | ✓ | ✓ | ✓ |  | 17 |
| Shortness of breath/difficulty breathing/shallow breathing | ✓ | ✓ |  |  | ✓ | ✓ | ✓ | ✓ | ✓ | ✓ |  | ✓ |  | ✓ | ✓ | ✓ | ✓ | ✓ | ✓ |  | 15 |
| Stuffy nose/nasal congestion |  | ✓ | ✓ |  | ✓ | ✓ | ✓ | ✓ | ✓ | ✓ |  |  | ✓ | ✓ | ✓ |  | ✓ | ✓ | ✓ | ✓ | 15 |
| Runny nose |  | ✓ | ✓ |  | ✓ | ✓ | ✓ | ✓ |  | ✓ |  | ✓ | ✓ |  | ✓ |  |  | ✓ | ✓ | ✓ | 13 |
| Body aches and pains |  |  |  |  | ✓ |  | ✓ |  | ✓ | ✓ | ✓ | ✓ | ✓ | ✓ | ✓ | ✓ |  | ✓ |  | ✓ | 12 |
| Interrupted sleep |  | ✓ | ✓ |  | ✓ |  | ✓ |  |  | ✓ | ✓ |  | ✓ |  | ✓ | ✓ | ✓ | ✓ | ✓ |  | 12 |
| Sore throat |  | ✓ | ✓ |  |  | ✓ | ✓ | ✓ | ✓ |  | ✓ |  | ✓ |  | ✓ | ✓ |  | ✓ | ✓ |  | 12 |
| Wheezing |  | ✓ |  | ✓ |  |  | ✓ | ✓ | ✓ |  |  | ✓ | ✓ | ✓ |  | ✓ |  | ✓ | ✓ | ✓ | 12 |
| Fever/feeling feverish/hot |  | ✓ |  | ✓ | ✓ | ✓ |  | ✓ |  | ✓ |  |  | ✓ | ✓ | ✓ |  |  | ✓ | ✓ |  | 11 |
| Mucous/productive cough |  | ✓ | ✓ | ✓ | ✓ | ✓ | ✓ |  |  | ✓ |  |  |  |  |  | ✓ | ✓ |  | ✓ | ✓ | 11 |
| Headache |  |  |  |  | ✓ |  | ✓ | ✓ | ✓ | ✓ | ✓ | ✓ | ✓ |  |  |  |  |  | ✓ | ✓ | 10 |
| Chest pain due to coughing/”sore ribs” |  | ✓ |  |  | ✓ |  | ✓ |  |  | ✓ |  |  |  |  |  | ✓ | ✓ | ✓ |  |  | 7 |
| Sounded “nasal-y”/voice changes/lost voice |  |  | ✓ |  |  | ✓ | ✓ |  |  | ✓ |  |  |  |  |  | ✓ |  |  |  |  | 5 |
| Chest tightness when trying to breathe |  | ✓ |  |  |  |  | ✓ |  | ✓ |  |  |  |  |  |  | ✓ |  |  |  |  | 4 |
| Sinus pressure/pain |  | ✓ |  |  |  |  | ✓ |  |  | ✓ |  | ✓ |  |  |  |  |  |  |  |  | 4 |
| Postnasal drip |  |  |  |  |  |  | ✓ |  | ✓ |  |  | ✓ |  |  |  |  |  |  |  |  | 3 |
| Sneezing |  | ✓ | ✓ |  |  |  |  |  |  | ✓ |  |  |  |  |  |  |  |  |  |  | 3 |
| Weakness/malaise |  |  | ✓ |  | ✓ |  |  | ✓ |  |  |  |  |  |  |  |  |  |  |  |  | 3 |
| Bronchospasm |  | ✓ |  |  | ✓ |  |  |  |  |  |  |  |  |  |  |  |  |  |  |  | 2 |
| Diminished appetite |  |  |  |  |  |  |  | ✓ |  |  |  |  |  |  |  |  |  | ✓ |  |  | 2 |
| Ear pain |  | ✓ |  |  |  |  |  |  |  | ✓ |  |  |  |  |  |  |  |  |  |  | 2 |
| Head congestion |  | ✓ |  |  |  |  |  |  |  |  |  | ✓ |  |  |  |  |  |  |  |  | 2 |
| Lightheaded/dizzy/fainting |  |  |  |  |  |  |  |  |  |  |  |  |  |  |  |  |  | ✓ | ✓ |  | 2 |
| Acid reflux |  |  |  |  |  |  |  |  |  |  |  |  |  |  |  |  | ✓ |  |  |  | 1 |
| Chest congestion |  |  |  |  |  |  | ✓ |  |  |  |  |  |  |  |  |  |  |  |  |  | 1 |
| Decreased libido |  |  |  |  |  |  |  |  |  |  |  |  |  |  |  |  |  |  | ✓ |  | 1 |
| Loose stool |  |  |  |  |  |  |  |  |  |  |  |  |  |  |  |  |  |  | ✓ |  | 1 |
| Vomiting |  |  |  |  | ✓ |  |  |  |  |  |  |  |  |  |  |  |  |  |  |  | 1 |
| Daily activity/functional impacts | | | | | | | | | | | | | | | | | | | | | |
| Participate in sports, hobbies, exercise |  | ✓ |  | ✓ | ✓ | ✓ | ✓ |  |  | ✓ | ✓ |  |  |  |  | ✓ |  |  | ✓ |  | 9 |
| Missing work/school | ✓ | ✓ | ✓ |  | ✓ |  |  |  |  |  |  | ✓ |  |  |  | ✓ |  | ✓ | ✓ |  | 8 |
| Self-care (e.g., dress, bathe, prepare meals) |  |  | ✓ |  | ✓ | ✓ | ✓ | ✓ |  |  | ✓ | ✓ |  |  |  |  |  | ✓ |  |  | 8 |
| Leaving home |  |  | ✓ |  | ✓ |  |  | ✓ | ✓ | ✓ |  |  |  |  |  |  |  | ✓ | ✓ |  | 7 |
| Ability to concentrate |  | ✓ |  |  | ✓ | ✓ |  | ✓ |  |  |  |  |  |  |  |  |  | ✓ | ✓ |  | 6 |
| Decreased productivity |  | ✓ |  |  | ✓ | ✓ |  |  |  |  |  |  |  |  |  |  |  | ✓ |  |  | 4 |
| Relationships with others |  |  |  |  | ✓ | ✓ | ✓ |  |  |  |  |  |  |  |  | ✓ |  |  |  |  | 4 |
| Canceling plans (e.g., concert, bowling league) |  |  |  |  | ✓ | ✓ |  |  |  | ✓ |  |  |  |  |  |  |  |  |  |  | 3 |
| Getting out of bed |  |  |  |  |  |  |  |  | ✓ | ✓ |  |  |  |  |  |  |  | ✓ |  |  | 3 |
| Using stairs |  |  |  |  |  |  |  | ✓ |  | ✓ |  |  |  |  |  |  |  |  | ✓ |  | 3 |
| Ability to care for others |  |  |  |  |  |  |  |  |  |  |  |  |  |  |  |  |  | ✓ |  |  | 1 |
| Cleaning |  |  | ✓ |  |  |  |  |  |  |  |  |  |  |  |  |  |  |  |  |  | 1 |
| Yard work |  |  |  |  |  |  |  |  |  |  |  |  |  |  |  | ✓ |  |  |  |  | 1 |
| Emotional impacts | | | | | | | | | | | | | | | | | | | | | |
| Worried | ✓ | ✓ | ✓ |  |  | ✓ |  | ✓ | ✓ | ✓ | ✓ |  |  |  | ✓ |  |  |  | ✓ |  | 10 |
| Frustrated/annoyed/aggravated |  | ✓ | ✓ |  | ✓ | ✓ | ✓ |  |  |  |  |  |  |  |  | ✓ |  | ✓ | ✓ |  | 8 |
| Irritable |  |  |  |  |  | ✓ |  |  |  |  |  |  |  |  |  |  |  | ✓ |  | ✓ | 3 |
| Despair |  | ✓ |  |  |  |  |  |  |  | ✓ |  |  |  |  |  |  |  |  |  |  | 2 |
| Helpless |  |  |  |  |  |  |  |  |  | ✓ |  |  |  |  |  |  |  |  |  |  | 1 |
| Panic |  | ✓ |  |  |  |  |  |  |  |  |  |  |  |  |  |  |  |  |  |  | 1 |
